# Link Prediction in Criminal Networks: A Tool for Criminal Intelligence Analysis

**DOI:** 10.1371/journal.pone.0154244

**Published:** 2016-04-22

**Authors:** Giulia Berlusconi, Francesco Calderoni, Nicola Parolini, Marco Verani, Carlo Piccardi

**Affiliations:** 1 Università Cattolica del Sacro Cuore and Transcrime, Milano, Italy; 2 MOX, Department of Mathematics, Politecnico di Milano, Milano, Italy; 3 Department of Electronics, Information and Bioengineering, Politecnico di Milano, Milano, Italy; Universiteit Gent, BELGIUM

## Abstract

The problem of link prediction has recently received increasing attention from scholars in network science. In social network analysis, one of its aims is to recover missing links, namely connections among actors which are likely to exist but have not been reported because data are incomplete or subject to various types of uncertainty. In the field of criminal investigations, problems of incomplete information are encountered almost by definition, given the obvious anti-detection strategies set up by criminals and the limited investigative resources. In this paper, we work on a specific dataset obtained from a real investigation, and we propose a strategy to identify missing links in a criminal network on the basis of the topological analysis of the links classified as marginal, i.e. removed during the investigation procedure. The main assumption is that missing links should have opposite features with respect to marginal ones. Measures of node similarity turn out to provide the best characterization in this sense. The inspection of the judicial source documents confirms that the predicted links, in most instances, do relate actors with large likelihood of co-participation in illicit activities.

## Introduction

Criminal intelligence analysis aims at supporting investigations, e.g. by producing link charts to identify and target key actors. Law enforcement agencies increasingly use Social Network Analysis (SNA) for criminal intelligence, analyzing the relations among individuals based on information on activities, events, and places derived from various investigative activities [1–3]. SNA provides added value compared to more traditional approaches like link analysis, by enabling in-depth assessment of the internal structure of criminal groups and by providing strategic and tactical advantages. For instance, SNA can inform law enforcement officers in the identification of aliases during large investigations and in the collection of evidence for prosecution [2]. Furthermore, the network analysis of criminal groups under investigation may help identify effective strategies to achieve network destabilization or disruption [3, 4].

Given the sensitiveness and implications of criminal proceedings, criminal intelligence and investigations strive for achieving the most accurate representation of each case. Information gathering and selection are crucial steps, due to the implications of both type I (false positive) and type II (false negative) errors. A number of controls and procedural safeguards are in place to prevent false positives (i.e. wrong accusations). Investigators, prosecutors, and courts routinely deal with irrelevant information by discarding it throughout the proceedings and keeping only material useful to build a case [5]. Contrarily, the inherently covert nature of criminal activities makes investigations more vulnerable to false negatives (i.e. missing information), with very limited solutions available to the law enforcement agencies due to time and resource constraints.

Missing information is also the main challenge for SNA of criminal networks. Law enforcement data from wiretap or other investigative sources are inevitably incomplete. Criminals often use communication and protection methods to decrease the effectiveness of law enforcement action [6]. Investigators rely on data-gathering methods, e.g. observations, archives, informants, witnesses, that results in incomplete information and thus a partial vision of the network under investigation [5, 7–9]. The lack of data generates problems of uncertain information, potentially jeopardizing the effectiveness of the investigations [3]. In the analysis of criminal networks, missing data can refer to missing nodes and/or missing links [7].

Missing nodes often depend on the scope and focus of the investigations. In turn, these may affect the specification of network boundaries, i.e. the definition of rules of inclusion of actors and their relations in the network [10–12]. Law enforcement agencies may overlook some important actors, especially if they take precautions against detection [5]. Research has shown that some skilled criminals assume a strategic position in criminal networks by balancing security and active involvement. Whereas intensive interaction with others normally increases the criminals’ performance, it also affects their visibility and consequently the vulnerability to law enforcement targeting. Some key players (e.g. the boss in a mafia) will avoid direct involvement in the illicit activities to reduce the risk of identification and arrest [13–15]. Nevertheless, the literature points out that even the most skilled criminals may hardly avoid detection in long lasting and intensive investigations, particularly if they have an important role in a criminal group [15].

Missing links instead refer to the lack of information on the relations between two known criminals. The police may miss meetings, conversations, and plans about criminal activities [5, 16]. For instance, criminals may use different telephone lines, according to the nature of the conversation and the interlocutor, and investigators may be able to identify only some of them. The frequent change of mobile phones and SIM cards and the use of particular lines to communicate with high-ranking affiliates may also prevent law enforcement agencies from identifying all conversations among suspects [17]. This results in incomplete information which may hinder or mislead investigations. Scholars and practitioners in criminology and criminal justice have often acknowledged the problem of missing links [5, 7, 16, 18]. Yet, studies on the their identification in criminal networks are still rare [19–21]. This is surprising, not only given the significant growth of works on missing links in other fields with the development of a number of different strategies [22–26], but also given that criminal investigations face the problem of missing links almost by definition, due to the scarcity of investigative resources and the anti-detection strategies by criminals [20].

This paper proposes an innovative strategy to identify possible missing links in a criminal network. It draws from the literature on link prediction and applies it on a unique dataset based on a real investigation. Differently from previous studies, the main assumption is that missing links may have characteristics contrary to those of marginal links discarded during the investigation. Indeed, while some links are ordinarily removed from a criminal network due to their marginality, other links with opposite characteristics may be missing due to lack of information. The analysis thus infers missing links *a contrario* from the characteristics of marginal links actually removed throughout the proceedings. The possible missing links so detected are highly probable social ties whose existence should be investigated by law enforcement agencies. Their identification during ongoing investigations may support law enforcement agencies in the allocation of scarce investigative resources, especially in the case of large criminal networks, and therefore improve the law enforcement action.

## Methods

### The Oversize dataset

The analysis relies on a unique dataset from operation Oversize, an Italian criminal case against a mafia group. The investigation lasted from 2000 to 2006, and targeted more than 50 suspects involved in international drug trafficking, homicides, and robberies. The trial started in 2007 and lasted until 2009, when the judgment was passed, and the main suspects were convicted with penalties from 5 to 22 years of imprisonment. Most suspects were affiliated to the ‘Ndrangheta, a mafia from Calabria (a southern Italian region) with ramifications in other regions and abroad [27, 28].

Contrarily to most empirical studies on criminal networks, which rely on data derived from a single source of information, Oversize’s peculiarity lies in the availability of three networks from three judicial documents corresponding to three different stages of the criminal proceedings [16]: the wiretap records (WR), the arrest warrant (AW), and the judgment (JU). The wiretap records include all wiretap conversations transcribed by the police and considered relevant at first glance. The arrest warrant contains a selection of the transcripts and other relevant information from informants and other investigative activities (e.g. physical surveillance). The judgment summarizes the trial and includes information from several sources of evidence, including wiretapping and audio surveillance. It is worth mentioning that the documents related to the arrest warrant and judgment are public [29, 30], whereas wiretap records are not publicly available because they report private conversations involving people other than suspects (access was obtained by the authors through a special permission). Nonetheless, the three networks, derived from a thorough, exhaustive analysis of the textual judicial documents [16], can be made public because no personal or sensitive information is reported (see Data Availability Statement).

Most studies on criminal networks focus on one or a small number of case studies, and rely on a single source of information [4, 7, 16, 18, 28, 31], because access to data is difficult to obtain, particularly in the case of wiretap records. The main limitation of a case study approach concerns the external validity of the findings, i.e. the extent to which the results can be generalized beyond the case studies [32]. The analysis of the Oversize dataset focuses on a single criminal network thus sharing similar limitations on external validity with previous studies. The peculiarity of the dataset (i.e. the availability of three networks) prevents replication on other cases. Yet, it simultaneously constitutes the strength and innovation of the current study because it enables observation of the discarded marginal links and the prediction of possible missing links.

The individuals involved in illicit activities constitute the nodes of the networks, the links indicate a relation between any two actors. We restrict the analysis to the undirected case, i.e. we neglect the directionality of links. The three networks are formally defined by *N*_*i*_ = (*V*, *E*_*i*_), *i* = *WR*, *AW*, *JU*, where *V* is the set of nodes (the same for all networks, with |*V*| = 182 nodes) and *E*_*i*_ is the set of links of network *i*. We denote by (*x*, *y*), with *x*, *y* ∈ *V*, any pair of nodes of network, be they connected by a link, i.e. (*x*, *y*) ∈ *E*_*i*_, or not. The number *c*_*xy*_ of telephone calls recorded between individuals (nodes) (*x*, *y*) is available for all (*x*, *y*) ∈ *E*_*i*_. Table 1 summarizes the main statistics of the three networks. We recall that the degree *k*_*x*_ of a node *x* is the number of links incident to *x*, i.e. the number of neighbor nodes. A node is isolated if *k*_*x*_ = 0. The density of the network is the ratio between the number of existing links |*E*_*i*_| and their maximum possible number |*V*|(|*V*| − 1)/2.

**Table 1:** Statistics of the Oversize networks.

	Wiretap Records	Arrest warrant	Judgement
n. of nodes (|*V*|)	182	182	182
n. of isolated nodes	0	36	93
n. of links (|*E*_*i*_|)	247	189	113
density	0.015	0.011	0.007
average degree	2.7	2.1	1.2
max degree	32	29	13

**Fig 1:**
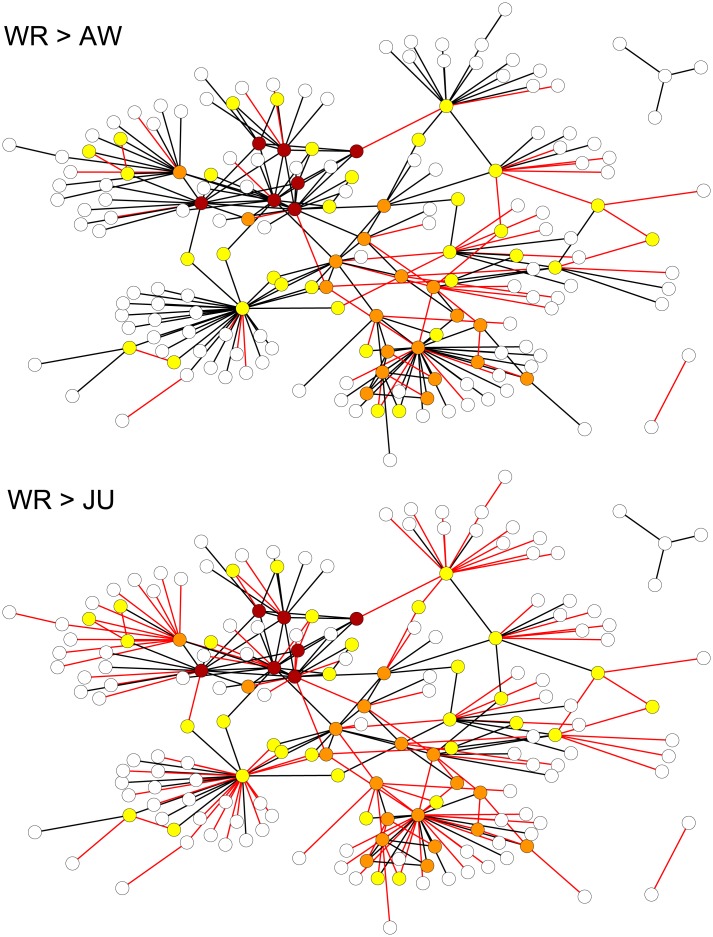
The Oversize networks. The links removed in passing from *N*_*WR*_ (Wiretap Records) to *N*_*AW*_ (Arrest Warrant) (*above*), or from *N*_*WR*_ to *N*_*JU*_ (Judgement) (*below*), are highlighted in red. Nodes are colored according to their coreness, based on the *k*-shell analysis of the *N*_*WR*_ network: 1 = white (most peripheral), 2 = yellow, 3 = orange, 4 = brown (most central).

The networks are simultaneously displayed in Fig 1. The Oversize networks show some features typical of illicit networks. Many criminal organizations analyzed in the literature exhibit the presence of a core of few highly-connected nodes and a large number of peripheral actors [4, 7, 28, 33–35]. Fig 1 highlights (through node coloring) the result of the *k*-shell core-periphery analysis [36]: nodes are partitioned into “concentric” layers (or shells), starting from the periphery and arriving to the core of the network. Each node is assigned to a shell: the 1-shell contains the most peripheral nodes, the 2-shell those which are in the layer immediately more internal, and so on. More in detail, the algorithm for *k*-shell decomposition can be summarized as follows [36]: put in the 1-shell (and remove) all nodes with degree *k*_*x*_ = 1, and then all nodes having *k*_*x*_ ≤ 1 after removal of the former; put in the 2-shell (and remove) all nodes with *k*_*x*_ = 2, and then all nodes having *k*_*x*_ ≤ 2 after removal of the former; etc. The procedure stops when all nodes have been classified in a *k*-shell. In the *N*_*WR*_ network, 4 shells are identified: they include, from the periphery to the core, 123, 33, 19, and 7 nodes, respectively, thus confirming the presence of a core of few actors and a large number of peripheral individuals.

### Network reduction and marginal links

In passing from *N*_*WR*_ to *N*_*AW*_, 58 of the 247 links of *N*_*WR*_ are removed (thus *E*_*WR*_ ⊃ *E*_*AW*_) creating 36 isolated nodes. Similarly, in passing from *N*_*WR*_ to *N*_*JU*_, 134 links are removed (*E*_*WR*_ ⊃ *E*_*JU*_) creating 93 isolated nodes. However, the links of *N*_*JU*_ are not a subset of those of *N*_*AW*_, i.e. the two reductions are not in cascade. This is normal, as subsequent phases of the criminal proceedings may generate new information, e.g. from witnesses or additional investigative activities.

**Fig 2:**
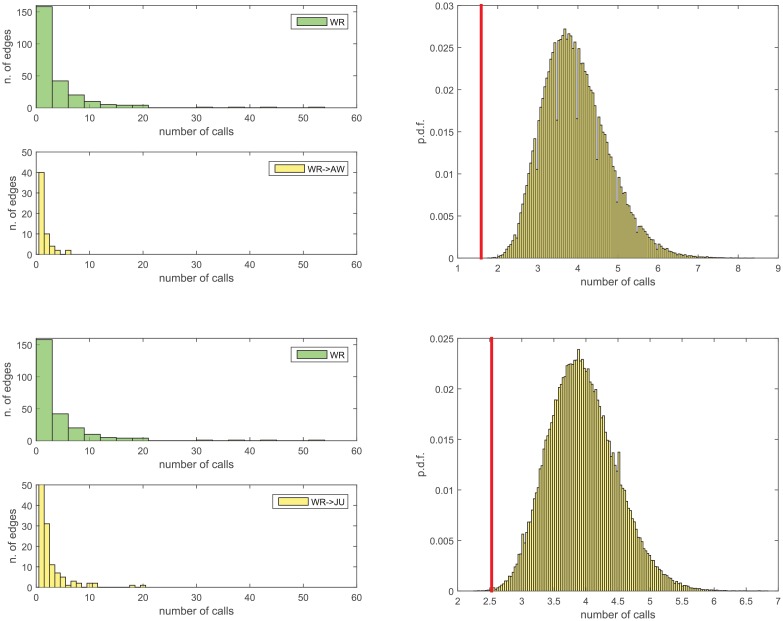
Statistics on the number of calls *c*_*xy*_ of the removed links. *Left panels*: in green, the histogram of the number of calls of the links of *N*_*WR*_. In yellow, the number of calls of the links removed in passing from *N*_*WR*_ to *N*_*AW*_ (*above*, 58 removals), and from *N*_*WR*_ to *N*_*JU*_ (*below*, 134 removals). *Right panels*: the distribution of the average number of calls of a random sample of 58 links (*above*) or 134 links (*below*) of *N*_*WR*_, compared with the average number of calls (red vertical line) of the links actually removed from *N*_*WR*_ to *N*_*AW*_ (*above*) and from *N*_*WR*_ to *N*_*JU*_ (*below*).

Fig 1 highlights the links removed in the network reduction processes (i.e. from *N*_*WR*_ to *N*_*AW*_, and from *N*_*WR*_ to *N*_*JU*_). The removed links are in most cases associated to a small number of telephone calls (Fig 2). In the original network *N*_*WR*_, the number of calls *c*_*xy*_ ranges from 1 to 52, with average value 〈*c*_*xy*_〉 = 3.95. On the other hand, the sets of removed links have 〈*c*_*xy*_〉_*WR* → *AW*_ = 1.59 (ranging from 1 to 6) and 〈*c*_*xy*_〉_*WR* → *JU*_ = 2.53 (from 1 to 20). None of the links with highest number of calls is removed. To substantiate this observation, we repeatedly select at random (for 10^5^ repetitions) 58 or 134 links from *N*_*WR*_, namely the same number of links removed, respectively, from *N*_*WR*_ to *N*_*AW*_ and from *N*_*WR*_ to *N*_*JU*_. It turns out (see the right panels in Fig 2) that the average number of calls of the links actually removed is extremely small, such that the probability of randomly selecting a smaller value is almost zero in both cases. It can safely be claimed that the link removal process tends to be biased by the intensity of the contacts between individuals, as the links with lower intensity are more likely to be removed.

The removed links often connect two individuals who had occasional contacts during the two-year investigation. In some cases, they concern pairs of actors who had telephone conversations in a few occasions and for very specific purposes (e.g. the purchase of small quantities of drugs). For instance, n63 (we refer to individuals by means of their anonymized label) was involved in a small number of telephone conversations with different retailers to arrange the purchase of small quantities of drugs in different occasions during the investigation. However, since he was not involved in the trafficking activities, nor in other serious crimes, the links between him and the other alleged criminals were discarded by the police when passing from *N*_*WR*_ to *N*_*AW*_. In other occasions, the links removed from the network involved at least one individual who had not been identified by the police. Indeed, 11 out of the 58 links discarded from *N*_*WR*_ to *N*_*AW*_ involved individuals who participated in (minor) illicit activities and are reported in the judicial documents with the initials “V.M.” (male voice) of “V.F.” (female voice), or with the name or nickname mentioned in the telephone conversations. The same applies to 32 links out of the 134 removed when passing from *N*_*WR*_ to *N*_*JU*_.

Other removed links with low intensity concern conversations about issues unrelated to the main illicit activities conducted by the members of the criminal group. Two examples are indicative of this type of links. In one occasion, n40 and n39 discuss about the debts that a third person has towards n39; in another occasion, n49 informs n26 of the arrest of another member of the group. In both cases, the links are formed as a consequence of an occasional communication between two individuals. Such communications may be useful to have a complete picture of the criminal network, but the links did not represent stable communication channels or relations among network members, nor they added any relevant information to the investigation process and they were discarded by the police.

Although it is certainly true that many removals involve peripheral nodes (especially in the *N*_*WR*_ to *N*_*JU*_ case), the visual inspection of Fig 1 reveals that many removals concern links which are instead connected, on one or both sides, to nodes with medium/large coreness. It cannot be claimed, therefore, that the network reduction is a process trivially involving the network periphery only. On the other end, we already pointed out that the intensity of the contacts (number of telephone calls) seems to be associated with the classification of marginal links by the police (see Fig 2). However, this quantity cannot be used for link prediction, since it cannot be associated in a straightforward way to a potential (non detected) link whose likelihood we want to quantify. As a matter of fact, there is no obvious way to associate a “predicted weight” to a predicted (thus non observed) link.

In the following, we will assess two topological indicators, namely link betweenness and node similarity, in their ability of characterizing the links which are marginal and thus, *a contrario*, in predicting the links which have not been detected but are likely to exist (missing links). These two quantities can indeed be used for this exercise, since their value can be naturally associated to a non existing (predicted) link, contrarily to the link weight (i.e. the number of calls). For this analysis, we will work on the unweighed (binary) network, i.e. we will neglect the information on the number of calls, both because we want to assess the predicting capabilities of the pure topological information (e.g. who is in contact with whom), and because the actual benefit of using weights in link prediction is known to be questionable [37].

#### Link betweenness

Our first hypothesis is that removed links are characterized by low betweenness. This means that they are redundant in the sense that they connect individuals who are already connected in some way in the network and do not significantly improve the flow of information. Networks are generally composed of subgroups (or communities) connected by one or a few links that bridge between them. “Structural holes”[38] are non-redundant contacts that lie in a brokerage position between otherwise disconnected components and thus facilitate the exchange of information and ideas. Links connecting different communities have high link betweenness (a generalization of Freeman’s node betweenness [39, 40]), since they are crucial to connect different parts of a network. Conversely, within-community links are to some extent redundant and their removal is likely to have little impact on the network. Our first hypothesis is thus tested through the computation of the link betweenness for both removed and non-removed links. We recall that, given a link (*x*, *y*) ∈ *E*_*i*_ connecting nodes *x* and *y*, the link betweenness *b*_*xy*_ is the number of shortest paths passing through (*x*, *y*), among those connecting all node pairs (*s*, *t*) of the network. More precisely:

$$\begin{array}{c} b_{xy}=\sum_{s,t\in V}\frac{B_{st}^{xy}}{B_{st}}, \end{array}$$ (1)

where *B*_*st*_ is the number of (equivalently) shortest paths connecting (*s*, *t*), and $B_{st}^{xy}$ is the number of such paths passing through (*x*, *y*). Betweenness thus emphasizes those links that favor the exchange of information among network members. The first hypothesis thus assumes that marginal links may have low betweenness and this may explain why they were discarded throughout the proceedings.

#### Node similarity

Our second, alternative hypothesis is based on the literature on link prediction. Several studies have applied different link prediction methods to a number of networks. They show that nodes are more likely to be connected when they are similar and share a number of features [22, 23]. According to the second hypothesis, thus, marginal links connect structurally dissimilar nodes, i.e. individuals who occasionally collaborate but are dissimilar in terms of interests, background, and involvement in criminal activities. Therefore, these connections are not crucial for the criminal conducts. The literature proposes several analytical strategies for link prediction, with new methods constantly added, mostly based on measures of node similarity [24–26]. Given the small size of the Oversize networks, such strategies are a viable option, since the exhaustive calculation of similarities for all node pairs is computationally feasible. The hypothesis is that marginal links have low similarity scores and this would explain their removal.

Node similarity approaches attribute a score *s*_*xy*_ to all node pairs (*x*, *y*) and, consequently, induce a ranking of all node pairs. Notice that, if (*x*, *y*) ∈ *E*_*i*_ (the set of links), *s*_*xy*_ can be interpreted as a score attributed to the link. Thus, node similarity actually yields a ranking of all the links *E*_*i*_. Among the many possible similarity scores, the simplest one amounts at counting the number of *Common Neighbors*(CN) of nodes (*x*, *y*):

$$\begin{array}{c} s_{xy}^{CN}=\left|\Gamma \left(x\right)\cap \Gamma \left(y\right)\right|, \end{array}$$ (2)

where Γ(*z*) denotes the set of neighbors of node *z*. The rationale is that (*x*, *y*) must have common features, interests, etc., if they have many common acquaintances. Thus it is likely that they are directly connected, or that they will in the near future. Empirical evidences of this assumption have been found in many instances [41, 42].

The CN similarity score can be refined in many ways, e.g. by weighting—not simply counting—the number of common neighbors. One of these ways leads to the definition of the *Resource Allocation* (RA) similarity score:

$$\begin{array}{c} s_{xy}^{RA}=\sum_{z\in \Gamma (x)\cap \Gamma (y)}\frac{1}{k_{z}}, \end{array}$$ (3)

where *k*_*z*_ = |Γ(*z*)| is the degree of node *z*. Here, the role of the common neighbor *z* in connecting (*x*, *y*) is diluted if *z* has many connections, since it will have less resources allocated to bridge (*x*, *y*).

CN and RA are widely used to quantify node similarity. Extensive tests on the capability of a broad set of indicators (including the two above) in solving the link prediction problem, found that CN obtains a very good performance despite its extreme simplicity, whereas RA ranks as one of the best indicators on a large set of benchmark tests [24].

## Results

**Fig 3:**
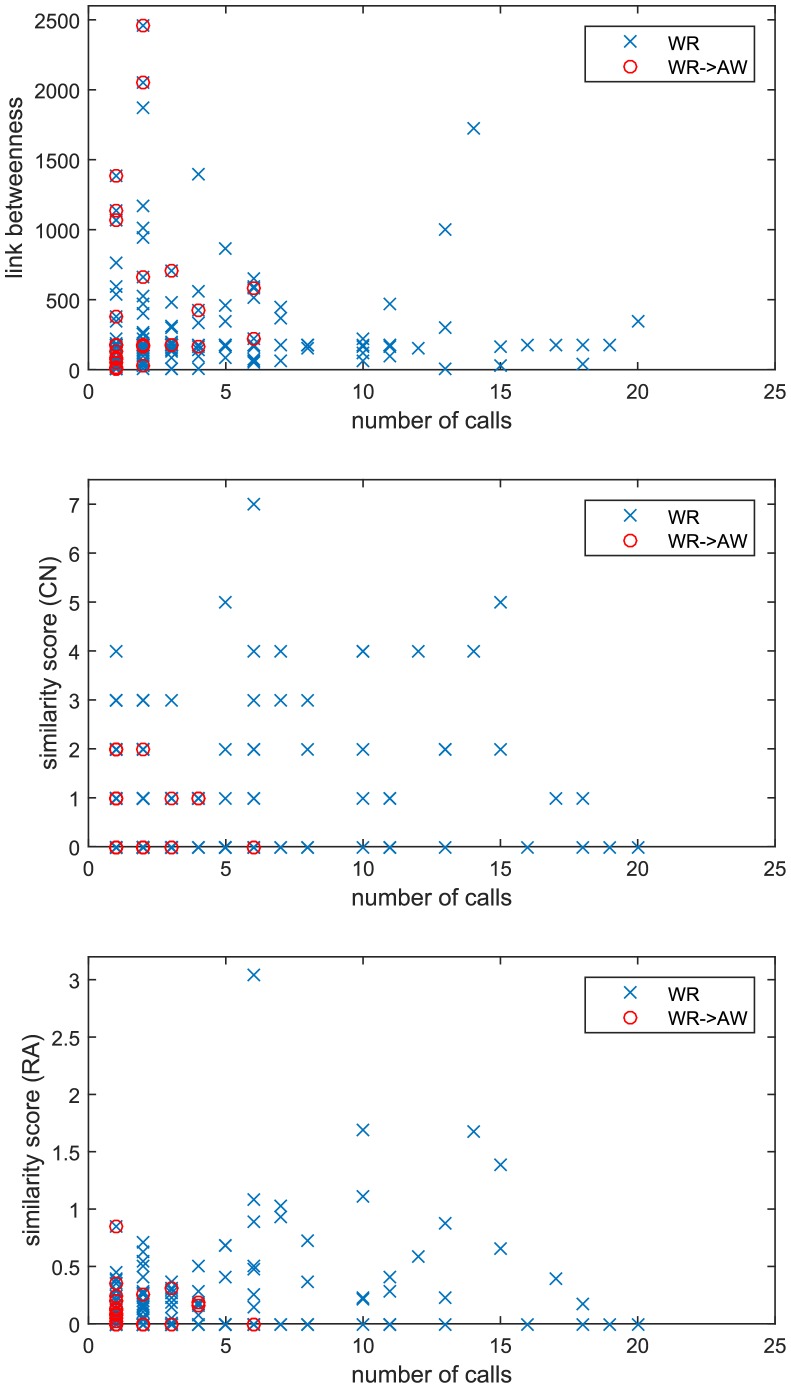
The relationship between the number of calls *c*_*xy*_, the link betweenness *b*_*xy*_, and the similarity score *s*_*xy*_ (CN and RA). Each blue cross corresponds to a link (*x*, *y*) ∈ *E*_*WR*_. Red circles highlight the links removed in passing from *N*_*WR*_ to *N*_*AW*_. The horizontal axis is truncated to improve readability: only 4 links over 247 have *c*_*xy*_ > 20, none of which is removed.

Fig 3 shows the relationship between the number of calls *c*_*xy*_, the betweenness *b*_*xy*_, and the similarity score *s*_*xy*_, both for the whole network *N*_*WR*_ and for the marginal links (here we only consider the reduction *N*_*WR*_ to *N*_*AW*_ for brevity). The figure reveals that all the removed links collocate among those with low similarity score, whereas we find removed links spread throughout the entire betweenness range. On the basis of this preliminary observation, we now consider the two hypotheses above discussed.

### Link betweenness

**Fig 4:**
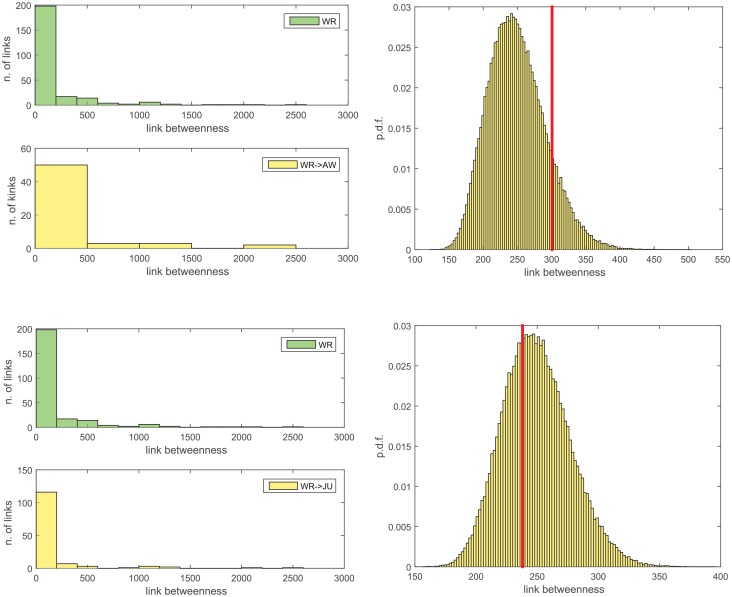
Statistics on the betweenness *b*_*xy*_ of the removed links. *Left panels*: in green, the histogram of the betweenness of the links of *N*_*WR*_. In yellow, the betweenness of the links removed in passing from *N*_*WR*_ to *N*_*AW*_ (*above*, 58 removals), and from *N*_*WR*_ to *N*_*JU*_ (*below*, 134 removals). *Right panels*: the distribution of the average betweenness of a random sample of 58 links (*above*) or 134 links (*below*) of *N*_*WR*_, compared with the average betweenness (red vertical line) of the links actually removed from *N*_*WR*_ to *N*_*AW*_ (*above*) and from *N*_*WR*_ to *N*_*JU*_ (*below*).

To check the first hypothesis (i.e. the removed links have low betweenness), we compute the betweenness of all links of the network *N*_*WR*_ and we compare their statistics to those of the links which are removed in passing to *N*_*AW*_ or, respectively, *N*_*JU*_. The results are summarized in Fig 4. The average betweenness of the links of *N*_*WR*_ is 〈*b*_*xy*_〉_*WR*_ = 249.4, and those of the removed links are not largely dissimilar, namely 〈*b*_*xy*_〉_*WR* → *AW*_ = 300.7 and 〈*b*_*xy*_〉_*WR* → *JU*_ = 238.0, respectively. Incidentally, some of the removed links have betweenness value of the order of the highest values found in the network (left panels in Fig 4). Furthermore, if we repeatedly select at random (for 10^5^ repetitions) 58 or, respectively, 134 links to remove (these are the number of links removed from *N*_*WR*_ to *N*_*AW*_ and, respectively, from *N*_*WR*_ to *N*_*JU*_), we discover that the average betweenness of the links actually removed is by no means anomalously small—in the *N*_*WR*_ to *N*_*AW*_ case it is even larger than average (right panels in Fig 4). This leads to the rejection of our first hypothesis.

### Node similarity

**Fig 5:**
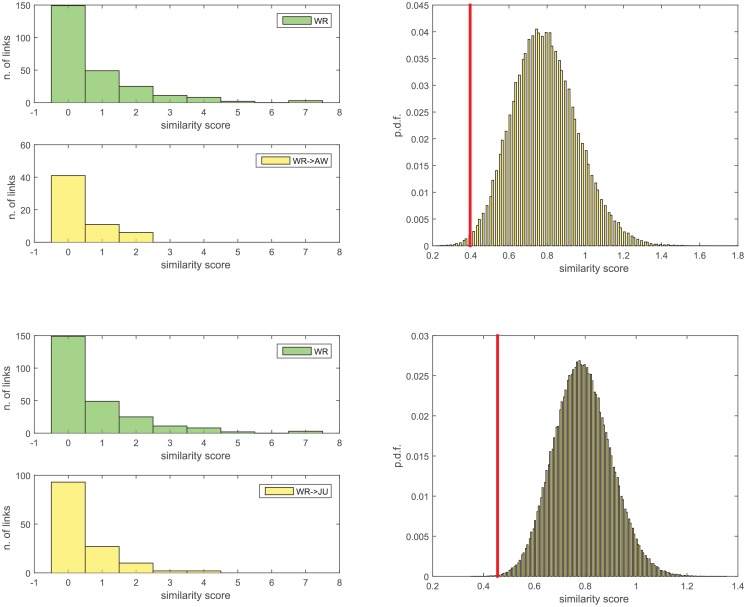
Statistics on the similarity score *s*_*xy*_ (CN) of the removed links. *Left panels*: in green, the histogram of the score of the links of *N*_*WR*_ (247 links). In yellow, the score of the links removed in passing from *N*_*WR*_ to *N*_*AW*_ (*above*, 58 removals), and from *N*_*WR*_ to *N*_*JU*_ (*below*, 134 removals). *Right panels*: the distribution of the average score of a random sample of 58 links (*above*) or 134 links (*below*) of *N*_*WR*_, compared with the average score (red vertical line) of the links actually removed from *N*_*WR*_ to *N*_*AW*_ (*above*) and from *N*_*WR*_ to *N*_*JU*_ (*below*).

We now move to our second hypothesis (i.e. the removed links connect structurally dissimilar nodes) and adopt a strategy common in the research on missing links, i.e. node similarity. We compute the similarity score *s*_*xy*_ (i.e. the similarity of the node pair (*x*, *y*)) of all the links of the network *N*_*WR*_, and we compare their statistics to those of the links which are removed in passing to *N*_*AW*_ or, respectively, *N*_*JU*_. The results are summarized in Fig 5 for the CN similarity score (Eq (2)). The average score of the links of *N*_*WR*_ is 〈*s*_*xy*_〉_*WR*_ = 0.789, whereas those of the removed links are much smaller, namely 〈*s*_*xy*_〉_*WR* → *AW*_ = 0.397 and 〈*s*_*xy*_〉_*WR* → *JU*_ = 0.455, respectively. None of the removed links has a score of the order of the highest values found in *N*_*WR*_ (left panels in Fig 5).

To give statistical significance to the above observation, we repeatedly select at random (for 10^5^ repetitions) the same number of links removed from *N*_*WR*_ to *N*_*AW*_ and, respectively, from *N*_*WR*_ to *N*_*JU*_ (58 or 134 links). The average score of the links actually removed is extremely small, such that the probability of randomly selecting a smaller average score is *p* < 0.01 in both cases (right panels in Fig 5). This means that the link removal process, if assessed in terms of similarity score *s*_*xy*_, appears to be strongly biased towards the links with least score. In this respect, the number of calls *c*_*xy*_ and the score *s*_*xy*_ associated to links seem to play a similar role in driving the removal process. However, as already pointed out, the former cannot be used for link prediction purposes.

The above results are confirmed if we instead adopt the RA similarity score (Eq (3)). Here the average score of the links of *N*_*WR*_ is 〈*s*_*xy*_〉_*WR*_ = 0.124, whereas those of the removed links are 〈*s*_*xy*_〉_*WR* → *AW*_ = 0.046 and 〈*s*_*xy*_〉_*WR* → *JU*_ = 0.067. Again, the probability of randomly selecting a smaller average score is *p* < 0.01 in both cases. Therefore, the hypothesis that removed links connect individuals who are structurally dissimilar (i.e. individuals who occasionally collaborate but are different in terms of tasks and involvement in criminal activities) can be accepted. Node similarity scores can thus be adopted to identify missing links within the Oversize network.

### Prediction of missing links

Our goal is now to identify the possible missing links in the Oversize network by inferring them *a contrario*, on the basis of the characteristics of the marginal links (i.e. links removed along the criminal proceedings) identified through the testing of the two hypotheses above. As a matter of fact, given that the link removal process proved to be strongly biased towards the smallest similarity scores, it is reasonable to presume that unobserved links (i.e. pairs of actors) with large similarity scores might be connected by missing links. In other words, if a small similarity between two actors—although connected—reveals the marginality of their link, a large similarity should be indicative of a connection, even when the link was not identified by law enforcement agencies. The procedure of attributing large likelihood of existence to links connecting highly similar nodes is at the basis of network reconstruction in all those fields where the knowledge of the complex set of interactions among agents is admittedly largely incomplete, such as for instance in social [12] or biological networks [43].

Let us first consider the CN score, defined by Eq (2). If we compute the similarity *s*_*xy*_ of the 247 links of the network *N*_*WR*_, we find that they range from 0 to 7, with average value 〈*s*_*xy*_〉 = 0.789. On the other hand, if we compute *s*_*xy*_ for all (*x*, *y*) ∉ *E*_*WR*_, i.e. for all node pairs not directly connected, we find values ranging from 0 to 5, but a much smaller average 〈*s*_*xy*_〉 = 0.123. Indeed, if we exhaustively consider all the combinations of a pair (*x*, *y*) ∈ *E*_*WR*_ with another (*x*, *y*) ∉ *E*_*WR*_, we find that the latter has a higher *s*_*xy*_ than the former in 19.7% of the cases only.

Since *s*_*xy*_ is significantly higher for pairs (*x*, *y*) directly connected, it is reasonable to presume that those pairs (*x*, *y*) ∉ *E*_*WR*_ with extremely large *s*_*xy*_ be actually connected by a missing link, i.e. a link existing but not experimentally observed. More precisely, if we set a threshold value *S* (typically large), we can compute the fraction *α* (typically small) of existing links (*x*, *y*) ∈ *E*_*WR*_ with *s*_*xy*_ ≥ *S*. If we now take a pair (*x*′, *y*′) ∉ *E*_*WR*_ such that *s*_*x*′ *y*′_ ≥ *S*, then the probability that *s*_*x*′ *y*′_ ≥ *s*_*xy*_ is larger than 1 − *α* (i.e. typically large) for whatever (*x*, *y*) ∈ *E*_*WR*_, namely the predicted link (*x*′, *y*′) collocates among the node pairs with higher similarity.

**Fig 6:**
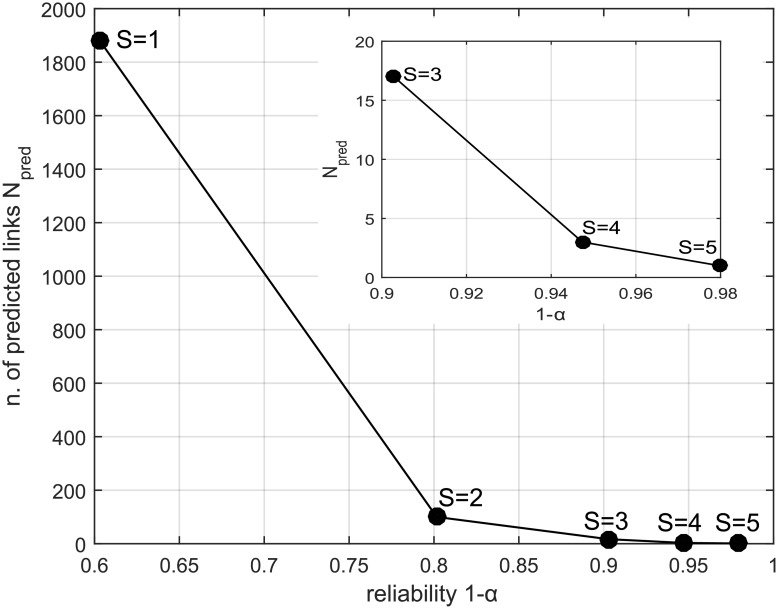
Link prediction with CN similarity score. The plot visualizes the relationship between the number of predicted links *N*_*pred*_, the link reliability 1 − *α*, and the similarity threshold *S*. The inset replicates the part of the plot with the highest reliability values.

**Fig 7:**
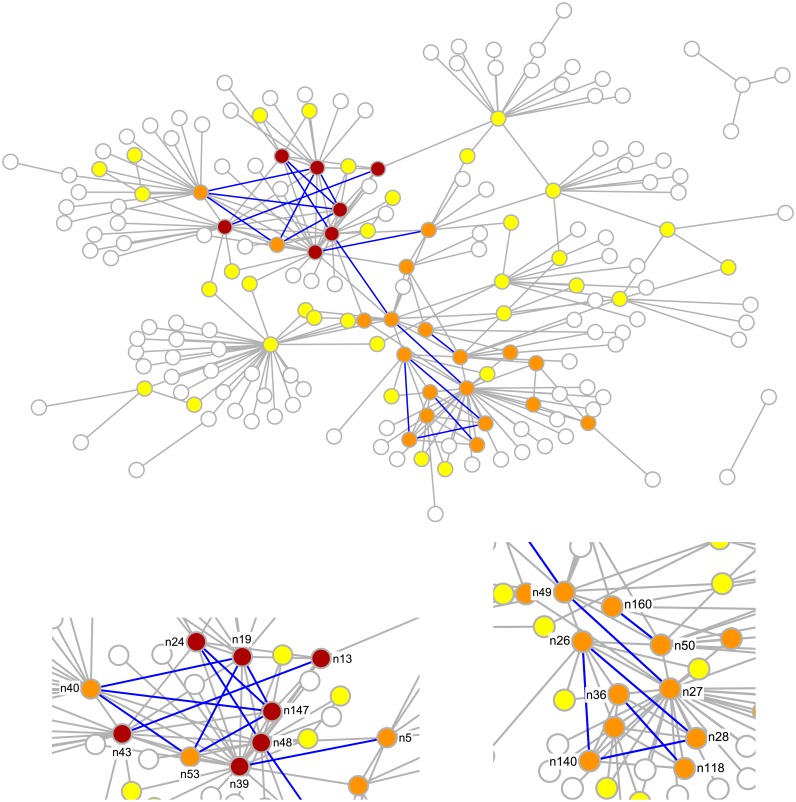
Link prediction. The Oversize network *N*_*WR*_ of the Wiretap Records (nodes and links in grey), with the 17 predicted links with largest CN similarity score *s*_*xy*_ (in blue). Nodes are colored according to their coreness based on the *k*-shell analysis (1 = white (most peripheral), 2 = yellow, 3 = orange, 4 = brown (most central)). The two parts of the network most relevant for link prediction are magnified in the bottom.

Fig 6 reports the relationship between the similarity threshold *S*, the number of predicted links *N*_*pred*_, and the link “reliability” 1 − *α*. In the following we focus our discussion on *S* = 3, a value which corresponds to 1 − *α* ≈ 0.90 and to a number of 17 predicted links (among the |*V*|(|*V*| − 1)/2 − |*E*_*WR*_| = 16224 pairs non directly connected). It is a reasonable trade off between a too tight (*S* = 4, with *N*_*pred*_ = 3) and a too loose threshold (*S* = 2, with *N*_*pred*_ = 100), as the number of predicted links is of the order of roughly one tenth of the existing links. The predicted links are highlighted in Fig 7. Notice that they mostly connect nodes with large centrality (i.e. *k*-shell coreness), and thus they could represent important, yet overlooked, relationships among key individuals.

In the light of that, we carried out a new campaign of analysis of the judicial documents to discover clues of the possible connections among the relevant individuals: the results are discussed below and summarized in Table 2. It should be emphasized that the absence of the predicted links from the original network *N*_*WR*_ essentially means that those connections have not corresponded to a recorded telephone call in the period of investigation (see the Discussion section for an overview of possible motivations). This does not exclude, however, the existence of a social connection of whatever nature, which is crucial to be discovered in order to have the most possible complete picture of the criminal network.

**Table 2:** The 17 predicted links with largest CN similarity score, with the specification of the role of the individuals involved. For all predicted links, with the only exception of (n5, n39), the analysis of the judicial documents finds evidence of the likelihood of a social tie.

predicted link (*x*, *y*)	node *x*	node *y*
(n49, n27)	boss’ son and important drug dealer	important drug dealer
(n49, n48)	boss’ son and important drug dealer	drug wholesaler
(n50, n160)	n49’s brother and drug dealer	fugitive and broker
(n118, n36)	n45’s wife	drug dealer
(n13, n43)	drug dealer	drug dealer
(n40, n53)	drug retailer	drug retailer
(n40, n147)	drug retailer	drug retailer
(n53, n147)	drug retailer	drug retailer
(n19, n40)	n48’s boss and important drug dealer	drug retailer
(n19, n53)	n48’s boss and important drug dealer	drug retailer
(n19, n147)	n48’s boss and important drug dealer	drug retailer
(n24, n48)	n19’s assistant	drug wholesaler
(n24, n147)	n19’s assistant	drug retailer
(n28, n26)	n27’s younger brother	n27’s assistant and drug wholesaler
(n28, n140)	n27’s younger brother	n27’s assistant and drug wholesaler
(n26, n140)	n27’s assistant and drug wholesaler	n27’s assistant and drug wholesaler
(n5, n39)	’recruiter’ and drug dealer	drug wholesaler

Node similarity predicts a link between n49 and n27, two of the main traffickers within the criminal network; n49 is the son of the boss and, with his father in jail, he was in charge of the trafficking activities, the management of the criminal group, and the investment of the proceeds of crime in both legal and illegal activities; n27 was heavily involved in the drug trafficking activities; in particular, he was charged with being responsible of the purchase and retail of large quantities of cocaine. Considering their role within the criminal group, it is highly probable that the two knew each other personally and had contacts. Similar considerations apply to the missing link identified between n49 and n48, who was in charge of the wholesale distribution of the drug in the province of Lecco, in the north of Italy. The judicial documents suggest that they collaborated with the mediation of other members of the criminal organization. However, both n49 and n48 lived in the same area and had key roles in the drug distribution chain, increasing the likelihood of a link between the two, as identified by the node similarity scores.

Node similarity also predicts a link between n50 and n160. The former is n49’s brother, also involved in drug trafficking activities. The latter is a fugitive who acted as a broker in the wholesale of drug. His being on the run was favored by n49, who provided constant support to n160. Considering the strong link between n49 and n160, and the close relationship between n49 and n50, it is likely that n50 and n160 also knew each other personally. Another predicted link is the one between n118, who is the wife of n45, and n36. Indeed, n118 is one of the few women suspected of being involved in the illicit activities of the criminal group. She was aware of her husband’s involvement in drug trafficking and her telephone calls discussing drug debts were intercepted by the police. The husband of n118 used to buy cocaine from n36 on behalf of other members of the criminal organization. The two men’s frequent contacts and n118’s involvement in illicit activities indicate that n118 and n36 may have known each other. The likelihood of a link between n13 and n43, also predicted by node similarity measures, is confirmed by a telephone call intercepted by Italian law enforcement agencies during the investigation. No conversations were recorded between the two alleged criminals; however, in June 2004 n13 informed another member of the organization of n43’s arrest, indicating that n13 and n43 knew each other.

Other links predicted by the CN similarity score include those forming a closed triad among n40, n53, and n147. The three suspects were involved in the drug retail in the province of Lecco and they used to buy the drug from the same wholesalers. As for n49 and n48, sharing drug distribution channels and operating in the same area justifies high node similarity scores. Nodes n40, n53, and n147 all share a missing link with n48’s boss n19, a drug trafficker involved in the wholesale of cocaine in the province of Lecco. A direct link between n19 and the three retailers was never confirmed by the police; however, the four suspects had trade relationships through n19’s subordinates, including n48, and they may have known each other personally. Two links were also predicted between n19’s assistant n24, and n48 and n147, respectively. The need to balance security and efficiency may have resulted in a division of labor between n19 and n24, with the former dealing cocaine with n48 and—indirectly—n147, and the latter having contacts with other wholesalers and retailers in the Lecco province. The strong relationship between n19 and n24, however, makes the predicted links very likely to have existed in the criminal organization. Another closed triad is formed by predicted links among n28, n26, and n140: as a matter of fact, n28 is n27’s younger brother; his activities included blending and hiding cocaine before its sale. The drug was then distributed by n27 with the help of n26, n140 and other wholesalers. Although no conversations or meetings were recorder among n28, n26, and n140, it is thus likely that they knew each other or had contacts in the past.

Overall, the thorough analysis of the judicial documents allowed us to validate, with a reasonable degree of reliability, 16 out of 17 of the links predicted by the CN similarity scores.

We now move to investigating the predicting capabilities of the RA similarity score, defined by Eq (3). The relationship between the similarity threshold *S*, the number of predicted links *N*_*pred*_, and the link “reliability” 1 − *α* is not only qualitatively, but also quantitatively very similar to that displayed in Fig 6 for the CN score (we omit the figure for the sake of conciseness). In particular, to facilitate a direct comparison with the CN results, we select again a threshold value (in this case *S* = 0.45) such that 17 links are predicted with a reliability 1 − *α* ≈ 0.90. It turns out that the links predicted by RA have only a partial overlap with those predicted by CN, since only 5 links out of 17 are designated by both methods. The attempt of validating the 12 new links through the analysis of the judicial documents, however, was not conclusive: no strong evidences were found for them, contrarily to what above described for the CN score.

**Fig 8:**
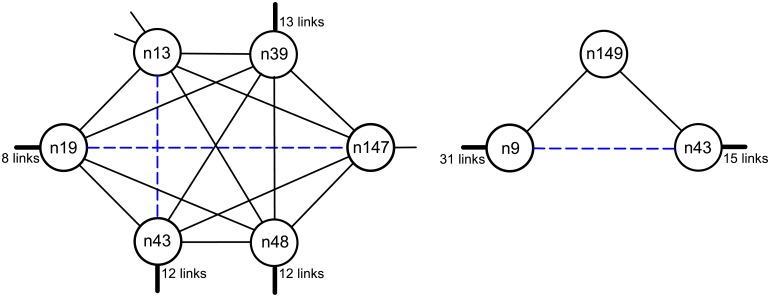
Examples of local topologies around predicted links. The left panel portrays the portion of the *N*_*WR*_ network around the link (n19, n147), predicted by the CN score (incidentally, (n13, n43) is also a predicted link). The right panel portrays the portion of network around the link (n9, n43) predicted by the RA score.

It seems therefore that the RA similarity score, in this specific case, has a weaker predicting capability than the CN score. With the aim of interpreting this fact, we focus on the 12 links predicted by RA but not by CN: notice that, having selected *S* = 3 for CN, they necessarily correspond to node pairs having exactly 1 or 2 common neighbors. Non connected pairs, i.e. (*x*, *y*) ∉ *E*_*WR*_, have a maximum RA score of about *s*_*xy*_ = 0.625. In view of Eq (3), to get a top-ranking RA score it is sufficient to have a common neighbor which is exclusive to the node pair (i.e. a degree 2 node) since this guarantees *s*_*xy*_ ≥ 0.5 (only 12 node pairs out of 16224 meet this inequality). This represents a peculiar form of connection, especially if we compare it with the typical scenario of CN top-ranking pairs, which are instead connected by 4 or 5 common neighbors. Fig 8 displays two representative cases of predicted links which are in the top ranking positions for CN and RA, respectively, but are not predicted by the other method. The local network structure appears to be strongly different: in the CN case, the predicted link is immersed in a dense community, contrarily to the RA case. Indeed, if we compute the average clustering coefficient of the nodes connecting the predicted links which are not in common between the two methods, we find *c*_*avg*_ = 0.431 for CN and *c*_*avg*_ = 0.090 for RA, a clear indication of a different local topology. On the other hand, the local topology around the link predicted by RA suggests that n149 is likely to have the peculiar role of brokering two important subnetworks (notice the large number of neighbors of n9 and n43). If it is so, it is not suprising that no direct connection should exist, as the intermediation is exerted precisely by n149.

To further explore which link prediction methods are appropriate in this specific case, we broaden the scope of the analysis by testing two additional methods, namely the *Katz index* similarity (e.g., [24]) and the *Structural Perturbation Method* (SPM) [25]. Both of them are global, i.e., the likelihood of a predicted link depends on the entire network. This is not the case for the CN and RA methods, which are based on a similarity score *s*_*xy*_ whose value only depends on the local structure of the network around (*x*, *y*).

Given an undirected, unweighed network with adjacency matrix *A*, the Katz index defines the similarity of nodes (*x*, *y*) by

$$\begin{array}{c} s_{xy}^{Katz}=\beta A_{xy}+\beta^{2}{\left(A^{2}\right)}_{xy}+\beta^{3}{\left(A^{3}\right)}_{xy}+\ldots ={\left[{\left(I-\beta A\right)}^{-1}-I\right]}_{xy}, \end{array}$$ (4)

where 0 < *β* < 1/*λ*_max_(*A*) to ensure convergence. By recalling that (*A*^*k*^)_*xy*_ is the number of paths of length *k* connecting (*x*, *y*), and noting that *A*_*xy*_ = 0 if the link (*x*, *y*) does not exist (which is the case when we quantify the likelihood of (*x*, *y*) for prediction), we interpret Eq (4) as a generalization of the CN score, since it considers the paths of all lengths connecting (*x*, *y*) instead of those of length 2 only, which are those passing through the common neighbors.

For the network *N*_*WR*_ we have *λ*_max_(*A*) = 7.07 and thus 0 < *β* < 0.141. To facilitate the comparison with the results above discussed, we select again the top-17 predicted links according to index Eq (4). It turns out that the 17 predicted links are the same as those of CN in the range 0 < *β* < 0.060, while for *β* = 0.100 the links predicted in common by Katz and CN reduce to 13 (but only 4 in common by Katz and RA). Interestingly, the 4 new links predicted by Katz (they are (n9, n39), (n13, n40), (n24, n40), (n43, n143)) are, in the CN ranking, in the set immediately below the top-17. Most notably, we were able to find in the judicial documents clear evidence of the likelihood of these social ties (we omit the details for brevity). Overall, we can safely claim that the results of the global link prediction method based on Katz similarity are consistent with those of the CN approach and, as such, they depart significantly from those obtained by the RA method.

The SPM considers the set of predicted links as a perturbation of the nominal network (coded by the adjacency matrix *A*) which, however, preserves its structural features (see [25] for details). To quantify the sensitivity to perturbations, a small portion of links are randomly selected and removed, so that we can write *A* = *A*^*R*^ + Δ*A* with the (symmetric) matrix Δ*A* containing the removed links. Then *A*^*R*^ is decomposed according to its eigenbasis:

$$\begin{array}{c} A^{R}=\sum_{k=1}^{\left|V\right|}\lambda_{k}v_{k}v_{k}^{T}, \end{array}$$ (5)

where |*V*| is the number of nodes and *λ*_*k*_ and *v*_*k*_ are the eigenvalue of *A*^*R*^ and the corresponding orthogonal and normalized eigenvector, respectively. The perturbed matrix is obtained as

$$\begin{array}{c} \tilde{A}=\sum_{k=1}^{\left|V\right|}\left(\lambda_{k}+\frac{v_{k}^{T}\Delta Av_{k}}{v_{k}^{T}v_{k}}\right)v_{k}v_{k}^{T}, \end{array}$$ (6)

which can be interpreted as an approximation of *A* in a linear expansion based on *A*^*R*^. In practice, $\tilde{A}$ will be obtained as the average of many instances of Eq (6), each one computed for a different random removal Δ*A*. Finally, the predicted links (*x*, *y*) are those with largest ${\tilde{A}}_{xy}$ among the node pairs non connected in the original network, i.e., those with *A*_*xy*_ = 0.

If we apply the SPM to the adjacency matrix *A* of the network *N*_*WR*_, we find a set of top-17 predicted links which overlaps with that of the CN method by 11 to 14 links, according to parametrization (number of random removals and fraction of removed links). The links in common with RA, instead, are never more than 4. In all instances, the new links predicted by SPM turn out to be, in the CN ranking, in the set immediately below the top-17. As for the Katz index described above, the results of the SPM prediction are largely consistent with those of the CN approach and, on the contrary, depart significantly from those obtained by the RA method.

To summarize the results of the link prediction analysis, we have found three different methods (one local, CN, and two global, Katz index and SPM) whose results are largely overlapping. Most notably, these results find significant validation in the judicial documentation, since they correspond to social ties not included in wiretap records but nonetheless very likely to exist. On the other hand, the fourth method, RA, does not seem an appropriate tool for link prediction in this specific case: its results are divergent with respect to the other methods and, moreover, its predicted links cannot be validated through the available documents. Of course, the most general question on which other methods, among the many available [22–26, 44], are appropriate in this specific context remains open. However, our analysis indicates that a few methods able to provide reliable predictions do exist. Among them, CN should certainly be appreciated for its conceptual simplicity and easy computability.

## Discussion

The rejection of the first hypothesis, according to which marginal (i.e. discarded) links are those with low betweenness, has some interesting implications. From a network analysis standpoint, it is a fact that the criminal justice system discarded as marginal a number of links with high betweenness. This may appear surprising, as these links connected not only peripheral nodes but also nodes with medium-high coreness. Thus, they may appear to bridge the “structural holes” within the criminal group [38]. In fact, a careful analysis reveals that links with high betweenness include a few occasional contacts or communications unrelated to the illicit activities. Despite their apparent bridging function, from a criminal intelligence standpoint these links are marginal. Overall, we must conclude that link betweenness proved to be unable to discriminate between marginal and important links in the criminal network.

The second hypothesis, based on node similarity, performed definitely better in the identification of marginal links. The link removal process independently conducted by the criminal justice system focused on links with low similarity, whereas in all instances it considered as relevant those links with high similarity. This demonstrates that node similarity matters beyond the merely topological analysis, as we have evidence that it is also naturally embedded in the activities of the law enforcement agencies.

The specific nature of the criminal case and the design of the study prevent an exhaustive and conclusive verification of the predicted links. In this study, instead, it is possible to verify the prediction through independent analysis of the judicial sources. The information of the case shows that social ties corresponding to the predicted links are, in almost all instances, very likely to have existed in the criminal organization, although undetected by investigators and thus not annotated in the Oversize networks. Reasons for overlooking predicted links include suspects’ use of communication and protection methods, investigators’ limited time and resources, and reliance on imperfect data-gathering methods (e.g. covert observations, informants, witnesses)[5, 7–9]. It is also worth noting that strong empirical evidence from wiretaps or other investigative sources must be available to include a link between any two suspects in the judicial documents. Investigators may have suspected some of the predicted links without being able to demonstrate their actual existence. At the same time, since criminals face a trade-off between efficiency and security, they may have deployed several security strategies against law enforcement surveillance, thus impeding the detection of their interactions [13, 15].

## Conclusions

Previous studies suggest that various fields of law enforcement may benefit from SNA: identification of suitable targets for network destabilization and prediction of the impact of their removal; detection of aliases through the analysis of actors with similar patterns of connections; and identification of potential defectors according to their position in the network [2, 45]. In this paper, we show how SNA may support criminal intelligence analysis and ongoing investigations by identifying missing links among suspects.

This study demonstrated that node similarity, already applied in different fields for link prediction, can identify possible missing links also in criminal networks, when information is noisy or incomplete almost by definition. The criminal justice system deploys a number of guarantees against false positives such as incorrect accusations and interactions unrelated to criminal conducts. Conversely, effective strategies to prevent false negatives, such as missing information, are scarce. Due to constrained data collection resources, law enforcement agencies may indeed miss some actors and links, with negative consequences on intelligence and investigation activities. This applies to drug trafficking networks, such as the Oversize network, as well as to other types of covert networks including street gangs and terrorist groups. These criminal organizations can all be conceived as networks of relations among co-offenders based on kinship or criminal collaboration. Since the social network approach to crime focuses on the relationships among co-offenders rather than, e.g. their illicit activities [46], SNA can be used to analyze any type of criminal networks, from small and flexible groups of collaborating criminals to more structured organizations.

Node similarity measures helped identify the characteristics of the links independently removed throughout the criminal proceedings: the removal process was strongly biased towards the links with least node similarity score. This provided support to the hypothesis that links discarded by the investigators throughout the criminal proceedings connect individuals that are structurally dissimilar, i.e. they link individuals who occasionally collaborate but are dissimilar in terms of tasks and involvement in criminal activities. Therefore, the removed links are not crucial for the criminal conducts. Consequently, node similarity enabled prediction of links that are likely to exist, but that were undetected by the police. Missing links were inferred *a contrario* from the characteristics of removed links, on the assumption that pairs of unconnected actors with large node similarity scores were likely connected by missing links, but for several reasons went unnoticed by law enforcement agencies. Content analysis of the judicial sources independently corroborates the likelihood of predicted links. Moreover, the comparative analysis of different similarity scores reveals that not all of them have the same predictive capability: we argue that the reason lies in the different topological properties they highlight.

In conclusion, the results show that node similarity measures can inform ongoing criminal investigations. On one hand, the independent link reduction conducted by the law enforcement agencies confirms node similarity as an important property of relevant links. On the other hand, link prediction may point out where to direct the scarce investigative resources for more effective investigations or even uncover relevant patterns overlooked by law enforcement authorities, especially in the case of investigations targeting large networks or criminal organizations with sophisticated communication and protection methods. Besides their practical implications, the results extend the prediction of missing links to a field largely neglected so far.
